# Supplementary figures and images for: Differences in Bacterial Small RNAs in Stool Samples from Hypercholesterolemic and Normocholesterolemic Subjects
Source: Int J Mol Sci. 2023 Apr 13;24(8):7213. doi: 10.3390/ijms24087213 (PMC10138442; doi:10.3390/ijms24087213)

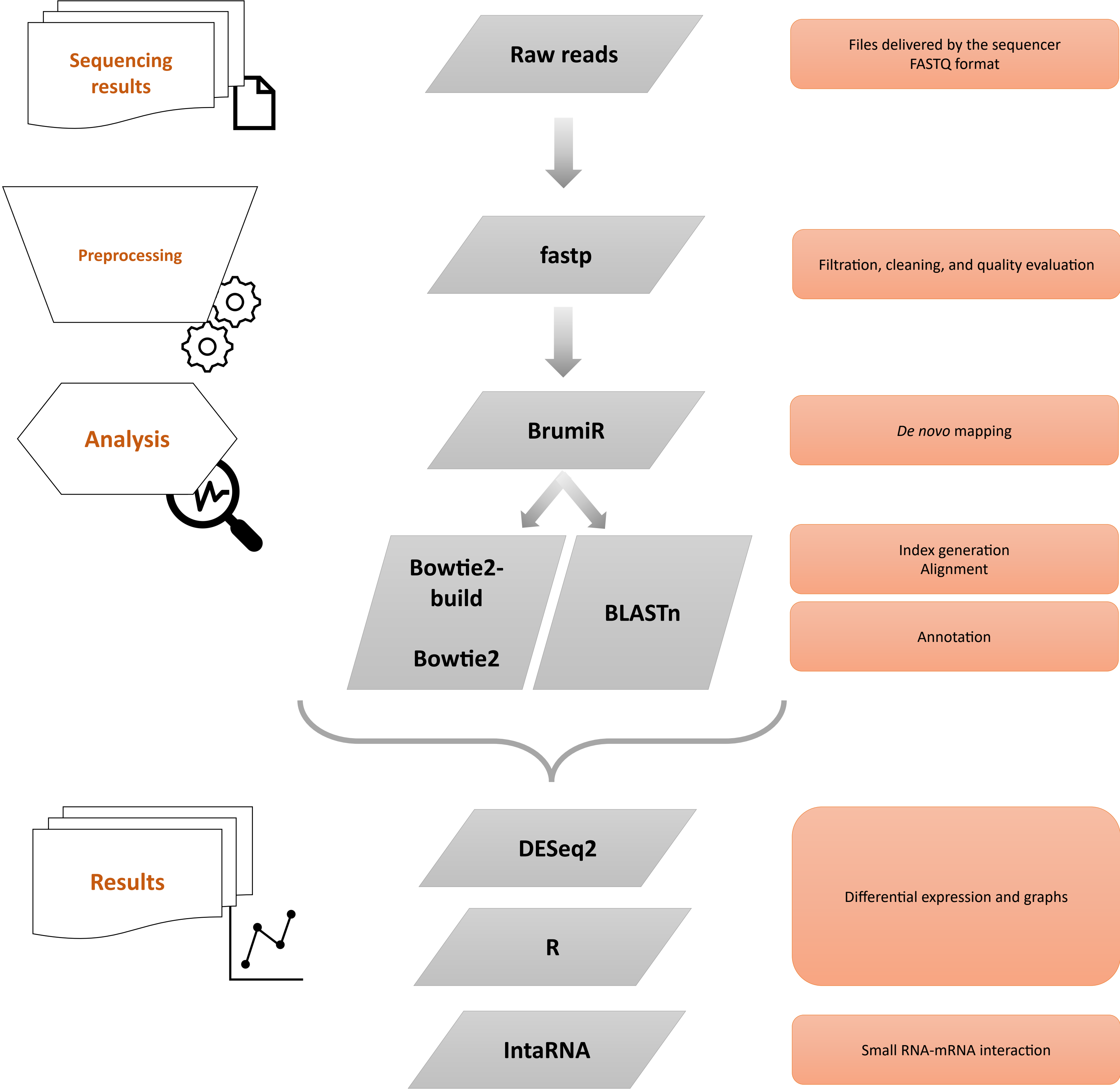

Supplement: Supplementary file 1 [file ijms-24-07213-s001.zip › supplementary FigureS1.pdf]
